# Probable Tigecycline‐Induced Acute Eosinophilic Pneumonia: A Case Report

**DOI:** 10.1002/rcr2.70726

**Published:** 2026-08-25

**Authors:** Annette Eom, Mutsumi Kioka

**Affiliations:** ^1^ Division of Pulmonary and Critical Care Medicine, Department of Internal Medicine Kirk Kerkorian School of Medicine, University of Nevada Las Vegas Nevada USA

**Keywords:** acute eosinophilic pneumonia, antibiotic‐induced lung injury, drug‐induced pulmonary toxicity, tigecycline

## Abstract

A 25‐year‐old man developed acute hypoxaemic respiratory failure after 21 days of tigecycline therapy for osteomyelitis caused by multidrug‐resistant organisms. Chest computed tomography demonstrated bilateral consolidations, ground‐glass opacities, interlobular septal thickening, small bilateral pleural effusions and mediastinal lymphadenopathy. Bronchoalveolar lavage (BAL) revealed marked eosinophilia (99%–100%) despite minimal peripheral eosinophilia. Alternative causes, including infectious, parasitic, autoimmune and other potential aetiologies, were carefully evaluated. Based on the temporal relationship with tigecycline exposure, marked BAL eosinophilia, exclusion of alternative causes and the overall clinical presentation, probable tigecycline‐induced acute eosinophilic pneumonia (AEP) was diagnosed. Tigecycline was discontinued, cefiderocol was initiated for concomitant multidrug‐resistant *Acinetobacter baumannii* , and systemic corticosteroid therapy was administered, followed by progressive clinical and radiographic improvement. Although the clinical response followed multiple simultaneous interventions and therefore does not establish causality, the overall findings support tigecycline as the probable causative agent. To our knowledge, this is the first reported case of probable tigecycline‐associated AEP. This case highlights the importance of considering AEP in patients who develop unexplained respiratory deterioration during tigecycline therapy and underscores the diagnostic value of BAL, particularly in the absence of marked peripheral eosinophilia.

## Graphical abstract

A 25‐year‐old man developed acute hypoxaemic respiratory failure after 21 days of tigecycline therapy for osteomyelitis caused by multidrug‐resistant organisms. Although the clinical response followed multiple simultaneous interventions and therefore does not establish causality, the overall findings support tigecycline as the probable causative agent. To our knowledge, this is the first reported case of probable tigecycline‐associated acute eosinophilic pneumonia (AEP) and highlights the importance of considering AEP in patients who develop unexplained respiratory deterioration during tigecycline therapy and underscores the diagnostic value of BAL, particularly in the absence of marked peripheral eosinophilia.

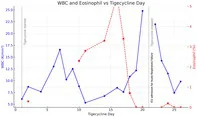

## 1 Introduction

Tigecycline is a semisynthetic glycylcycline antibiotic developed to overcome traditional tetracycline resistance. It has approximately fivefold greater binding affinity to bacterial ribosomes compared with earlier tetracyclines and is approved by the US Food and Drug Administration (FDA) for the treatment of complicated skin and soft tissue infections, intra‐abdominal infections and community‐acquired pneumonia. The most frequently reported adverse effects involve the gastrointestinal, hepatic and haematologic systems. In contrast, pulmonary toxicity is rare and remains poorly characterised [1].

Acute eosinophilic pneumonia (AEP) is an uncommon but potentially life‐threatening pulmonary disorder characterised by diffuse eosinophilic infiltration of the alveolar and interstitial spaces. Clinically, AEP presents with acute‐onset dyspnoea, hypoxaemia, fever and cough. Diagnosis typically relies on bronchoalveolar lavage (BAL) showing eosinophilia exceeding 25%, along with imaging that reveals bilateral pulmonary infiltrates. Notably, peripheral eosinophilia may be absent early in the disease course [2].

Although the aetiology of AEP is diverse, several medications—including antimicrobials such as minocycline—have been implicated as triggers, likely through immune‐mediated hypersensitivity reactions. Given its structural and pharmacologic similarity to minocycline, tigecycline may share a class effect, predisposing susceptible individuals to similar pulmonary complications. To our knowledge, no published reports of tigecycline‐associated AEP have been described previously. Here, we describe a case of probable tigecycline‐induced AEP, highlighting an important but underrecognised adverse effect of this broad‐spectrum antibiotic.

## 2 Case Report

A 25‐year‐old male with a complex medical history following traumatic injury presented with progressive bilateral toe gangrene requiring transmetatarsal amputation. His hospital course was complicated by osteomyelitis of the right second metatarsal, with wound cultures growing *Klebsiella pneumoniae* and *Candida auris* . Tigecycline (50 mg IV every 12 h) was initiated due to a history of infections with multidrug‐resistant organisms. The isolate represented an ESBL‐producing multidrug‐resistant *Klebsiella pneumoniae* , showing resistance to most β‐lactams but susceptibility to carbapenems, indicating an ESBL rather than carbapenemase‐mediated mechanism. The infectious disease team was consulted, and the culture results were considered to represent a true infection. Tigecycline therapy was initiated based on their recommendation.

**FIGURE 1:**
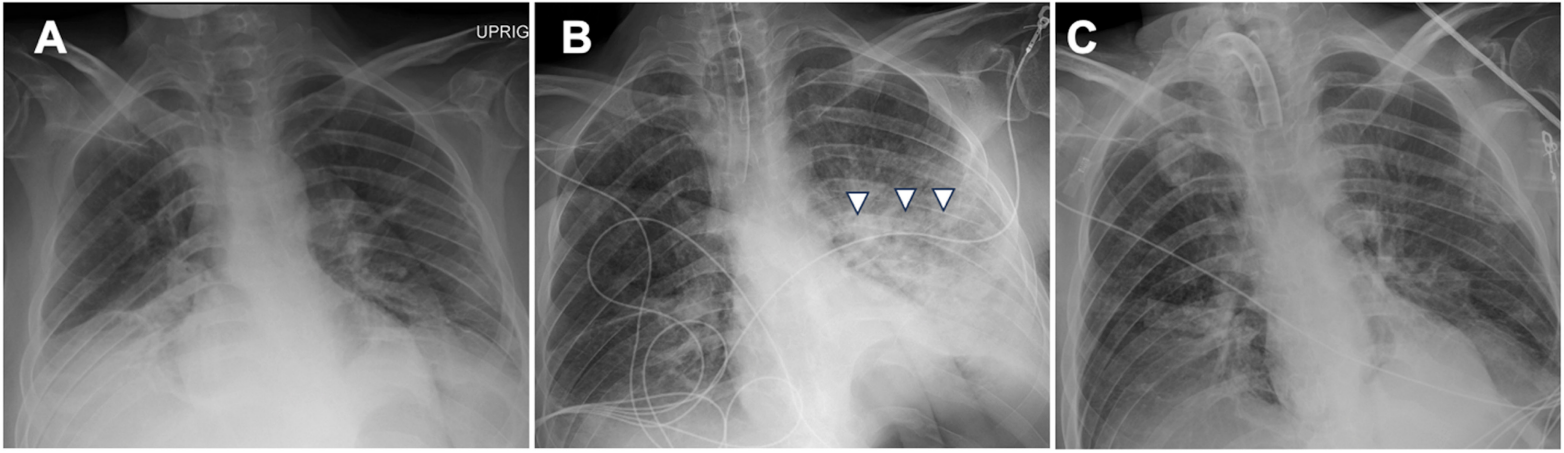
Serial chest radiographs showing the progression and subsequent improvement of bilateral pulmonary opacities and left‐predominant consolidation following tigecycline discontinuation, cefiderocol initiation and corticosteroid therapy. (A) Six days before tigecycline initiation, showing mild bilateral hazy pulmonary opacities. (B) On Day 21 of tigecycline therapy, demonstrating new dense left‐predominant consolidation, as indicated by white arrowheads. (C) The patient's respiratory status and imaging findings improved progressively over the following weeks, with radiographic improvement. He was discharged to a long‐term acute care hospital on 30 January for continued respiratory care and rehabilitation. The complete clinical course is summarised in Figure 3.

**FIGURE 2:**
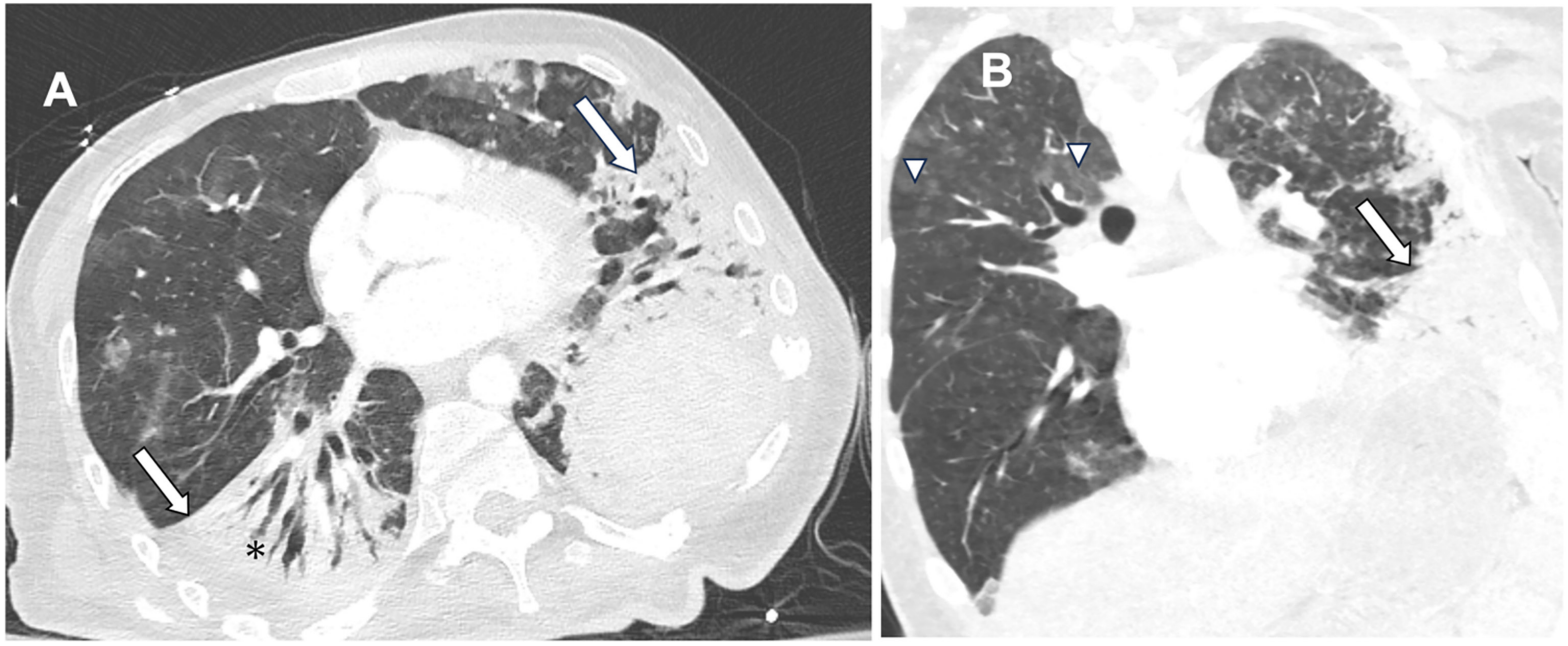
CT images obtained after 21 days of tigecycline therapy. (A) Axial view showing bilateral pulmonary consolidations with marked left‐sided predominance (white arrows), air bronchograms (*), small bilateral pleural effusions and mediastinal lymphadenopathy. (B) Coronal view demonstrating dense left‐predominant consolidation (white arrow), patchy ground‐glass opacities (white arrowheads) and interlobular septal thickening.

**FIGURE 3:**
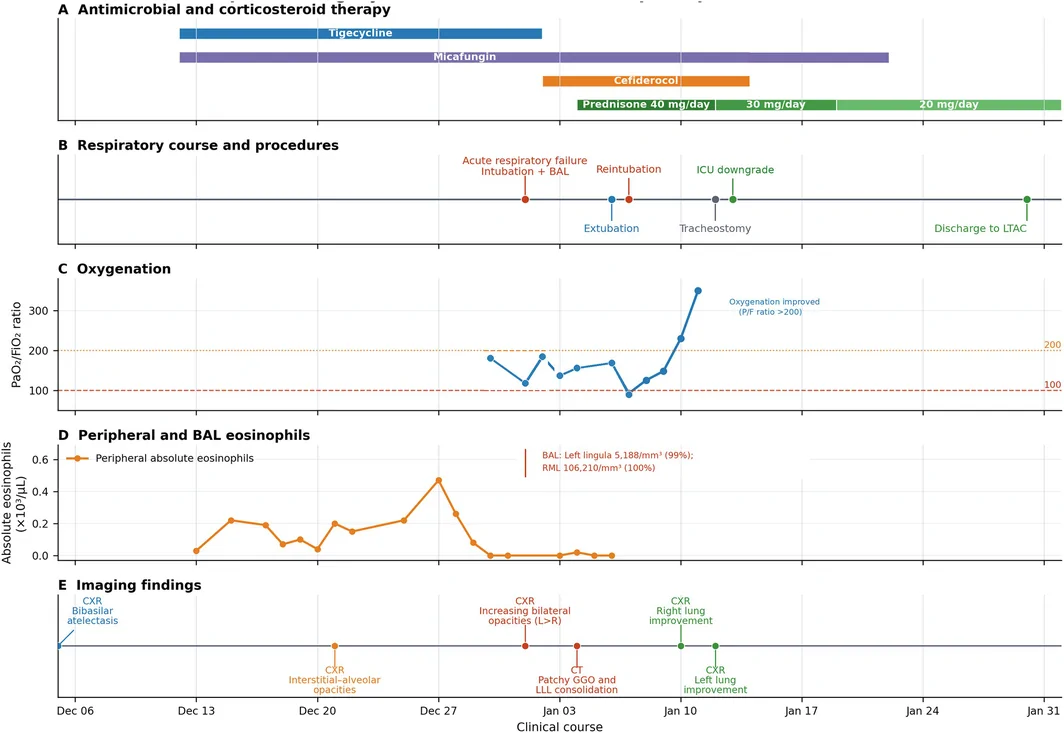
Comprehensive clinical timeline of probable tigecycline‐induced acute eosinophilic pneumonia. The timeline summarises the patient's clinical course from 6 December 2022 to 30 January 2023. (A) Duration of antimicrobial therapy and corticosteroid treatment, including tigecycline, cefiderocol and enteral prednisone taper. (B) Major clinical events and respiratory procedures, including acute hypoxaemic respiratory failure, bronchoscopy with BAL, intubation, extubation, re‐intubation, tracheostomy, ICU discharge and hospital discharge to a LTAC. (C) Oxygenation expressed as the PaO_2_/FiO_2_ (P/F) ratio. Dashed horizontal lines indicate P/F ratios of 100 and 200. (D) Peripheral absolute eosinophil count and BAL eosinophilia. BAL performed on 1 January demonstrated 99%–100% eosinophils. (E) Chronological chest imaging findings. The temporal relationship amongst tigecycline exposure, onset of respiratory failure, BAL eosinophilia, corticosteroid treatment and subsequent clinical improvement is illustrated. BAL, bronchoalveolar lavage; CT, computed tomography; CXR, chest radiograph; GGO, ground‐glass opacity; ICU, intensive care unit; LTAC, long‐term acute care hospital; P/F ratio, ratio of arterial oxygen partial pressure to fractional inspired oxygen; RML, right middle lobe.

**FIGURE 4:**
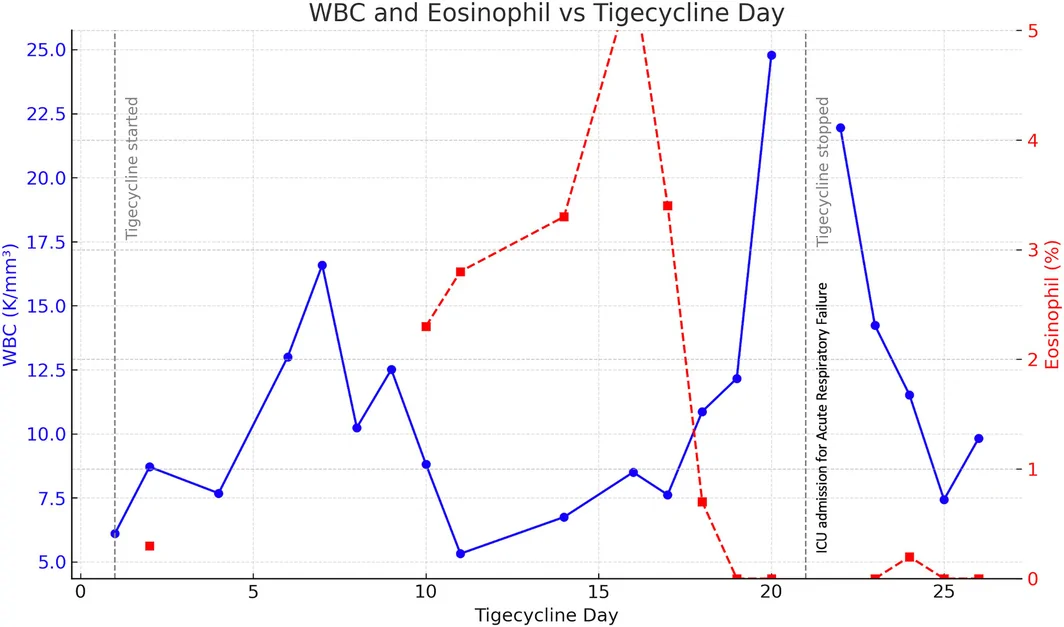
Temporal trends of white blood cell (WBC) count and peripheral eosinophil percentage during tigecycline therapy. The WBC count (blue line, left y‐axis) and eosinophil percentage (red dashed line, right y‐axis) are plotted against tigecycline treatment days. Tigecycline was initiated on Day 1 and discontinued on Day 21 following the onset of acute respiratory failure and ICU admission (vertical dashed lines). The WBC count exhibited a biphasic pattern, initially rising during the first week, transiently decreasing, and peaking again on Day 21 when acute respiratory failure occurred. In contrast, the peripheral eosinophil percentage peaked around Day 15–16 and subsequently decreased to 0% at the onset of respiratory failure. The figure presents the observed laboratory trends without assigning a specific mechanistic explanation.

Six days before tigecycline initiation, chest radiography (Figure 1A) demonstrated mild bilateral pulmonary opacities. The patient had been hospitalised since October following traumatic injuries and remained asymptomatic without hypoxaemia, fever or other respiratory symptoms. Given the prolonged hospitalisation and clinical stability, these mild opacities were considered most consistent with dependent atelectatic changes rather than an active pulmonary process. He had no history of asthma, atopy, allergic disease or previous eosinophilia. He was a never‐smoker with no recent cigarette exposure. Although he had a remote history of vaping before his traumatic injury, he remained continuously hospitalised thereafter, making recent vaping or other inhalational exposure unlikely. There was no reported inhalational recreational drug use, occupational, environmental or dust exposure or recent travel. On Day 18 of tigecycline therapy, coarse breath sounds were noted on examination, and the white blood cell (WBC) count reached the upper limit of normal (10.87 × 10^3^/μL); however, the primary team did not suspect pneumonia because the patient remained afebrile and without respiratory symptoms. Therefore, only incentive spirometry was prescribed for presumed atelectasis. On Day 21 of tigecycline therapy, the patient developed acute hypoxaemic respiratory failure requiring ICU admission and mechanical ventilation. Chest imaging revealed bilateral infiltrates (Figure 1B). CT angiography demonstrated bilateral consolidations and ground‐glass opacities with marked left‐sided predominance, as well as interlobular septal thickening, small bilateral pleural effusions and mediastinal lymphadenopathy (Figure 2). The PaO_2_/FiO_2_ (P/F) ratio was 118, consistent with moderate acute respiratory distress syndrome (ARDS). Bronchoscopy with BAL was performed in the lingula of the left upper lobe and right middle lobe before initiation of corticosteroid therapy; more specific segmental localization within these sampling sites was not documented. A total of 100 mL of sterile normal saline was instilled at each sampled site; however, the recovered volumes were not documented. BAL revealed marked eosinophilia (99%–100%) despite minimal peripheral eosinophilia (Table 1). The laboratory record documented a manual body‐fluid WBC calculation but did not specify the method used for the differential cell count or the number of cells counted. Review of the body‐fluid slide by a pathologist confirmed numerous eosinophils, although the available documentation did not indicate whether a cytospin preparation was used. Eosinophilic debris, Charcot–Leyden crystals and other specific cytologic findings were not documented. The reported BAL WBC count of 106,210/mm^3^ in the right middle lobe sample was verified against the original laboratory report. Targeted evaluation for alternative causes of eosinophilic lung disease, including fungal infection ( *Aspergillus* antigen testing), parasitic infection (stool ova and parasite examination), autoimmune disease (p‐ANCA and c‐ANCA) and respiratory viral infection (multiplex respiratory PCR performed on 8 January 2023), was negative. Total serum IgE was within the normal range (9 IU/mL). BAL culture demonstrated moderate semiquantitative growth of multidrug‐resistant *Acinetobacter baumannii* . Gram staining showed few WBCs, no epithelial cells and rare gram‐negative bacilli. BAL differential cell counts demonstrated marked eosinophilia (99%–100%) without a meaningful neutrophilic component, and intracellular organisms were not documented. Blood cultures obtained on 1 January and 8 January remained negative. Procalcitonin remained relatively low, increasing modestly from 0.16 ng/mL on 18 December and 0.17 ng/mL on 30 December to 0.22 ng/mL on 8 January. No previous microbiological records indicated prior colonisation with *A. baumannii* . The isolate was resistant to ampicillin/sulbactam, piperacillin/tazobactam, cefepime, meropenem, gentamicin, tobramycin, ciprofloxacin and amikacin, but remained susceptible to minocycline; interpretation of the tigecycline E‐test was unavailable. The Infectious Diseases team considered the BAL isolate to represent infection rather than colonisation, and cefiderocol was initiated because of the multidrug‐resistant susceptibility profile and limited therapeutic options. Serial chest radiographs obtained before tigecycline initiation demonstrated mild, nonspecific pulmonary opacities, including mild perihilar interstitial infiltrates on 7 October, increasing perihilar and bibasilar bandlike opacities on 14 November, and hypoexpanded lungs with trace bibasilar atelectatic changes on 5 December. At that time, the patient had no respiratory symptoms, hypoxaemia, fever or other clinical evidence of an active pulmonary process. After 21 days of tigecycline therapy, repeat imaging demonstrated marked progression with new diffuse bilateral infiltrates and ground‐glass opacities accompanying acute hypoxaemic respiratory failure. Given the marked BAL eosinophilia (99%–100%), temporal relationship with tigecycline exposure, radiographic progression and evaluation of alternative aetiologies, the acute respiratory failure was considered most consistent with probable tigecycline‐induced AEP, with concomitant multidrug‐resistant *A. baumannii* infection. Tigecycline was discontinued and cefiderocol was initiated for the multidrug‐resistant *A. baumannii* isolate. Enteral prednisone was initiated via a feeding tube on 4 January at 40 mg/day (approximately 1.1 mg/kg/day) and was subsequently tapered by 10 mg each week [3]. No intravenous corticosteroids were administered before initiation of enteral prednisone; the rationale for selecting the enteral route was not documented. The patient was extubated on 6 January after improvement and successful completion of a spontaneous breathing trial but required re‐intubation on 7 January because of recurrent respiratory failure. Oxygenation subsequently improved, with the P/F ratio increasing above 200 on 10 January. A tracheostomy was performed on 12 January, followed by ICU downgrade on 13 January. The patient's respiratory status and imaging findings improved progressively over the following weeks, with radiographic improvement shown in Figure 1C. He was discharged to a long‐term acute care hospital on 30 January for continued respiratory care and rehabilitation. The complete clinical course is summarised in Figure 3. The trends of WBC and peripheral eosinophil percentage in relation to tigecycline therapy are shown in Figure 4. The WBC count exhibited a biphasic pattern, initially increasing during the first week of therapy, transiently decreasing and subsequently peaking again on Day 21, coinciding with the onset of acute respiratory failure. The peripheral eosinophil percentage peaked at 5.5% (absolute eosinophil count 0.47 × 10^9^/L) around Day 15–16 of therapy and subsequently decreased to 0% at the onset of respiratory failure. These trends are presented descriptively, as the mechanisms underlying the changes in peripheral leucocyte and eosinophil counts could not be determined from this single case.

**TABLE 1:** Bronchoalveolar lavage (BAL) cell count with differential from samples obtained from the left lingula and right middle lobe.

Cell types	Sample 1 (left lingula)	Sample 2 (right middle lobe)
WBC count (/mm^3^)	5188	106,210
RBC count (/mm^3^)	< 2000	10,000
Neutrophils (%)	0	0
Lymphocytes (%)	0	0
Monocytes (%)	1	0
Eosinophils (%)	99	100
Basophils (%)	0	0

*Note:* The predominance of eosinophils (> 99%) is consistent with acute eosinophilic pneumonia.

## 3 Discussion

Eosinophilic lung diseases comprise a heterogeneous group of conditions characterised by eosinophilic infiltration of pulmonary tissue. Amongst these, AEP is a rapidly progressive disorder classified as a subtype of secondary eosinophilic lung disease. It typically presents with acute respiratory symptoms and marked eosinophilia in BAL fluid. According to the modified Philit criteria, the diagnosis of AEP is based on: (1) an acute respiratory illness of ≤ 1 month duration, (2) pulmonary infiltrates on imaging, (3) BAL eosinophilia > 25% and (4) exclusion of other eosinophilic lung conditions.

Peripheral eosinophilia may be absent in the early stages, which underscores the diagnostic importance of BAL. Imaging findings often include bilateral ground‐glass opacities, interlobular septal thickening and mediastinal lymphadenopathy. In cases of drug‐induced AEP, the diagnosis is supported by: (1) recent exposure to a known or suspected causative agent, (2) a temporal association with symptom onset, (3) exclusion of alternative diagnoses and (4) clinical improvement following drug discontinuation (2).

The present case met these diagnostic criteria and demonstrated a characteristic clinical course compatible with drug‐induced AEP. As illustrated in Figure 4, the patient's WBC count exhibited a biphasic trend during tigecycline therapy, with an initial increase, a transient decline and a subsequent peak on Day 21 coinciding with the onset of acute respiratory failure. However, the mechanisms underlying these changes cannot be established from this single case. The observed pattern may have been influenced by multiple factors, including the underlying infection, physiological stress, medication effects and the evolving pulmonary inflammatory process. Therefore, the leucocyte trends should be interpreted as descriptive observations rather than evidence of a specific biological mechanism [4].

Mechanistically, drug‐induced AEP is believed to result from a hypersensitivity reaction characterised by T‐helper 2 (Th2)–mediated immune activation, cytokine release, including interleukin‐5, interleukin‐4, interleukin‐13 and subsequent eosinophil recruitment into the pulmonary parenchyma [4]. Tigecycline is structurally related to minocycline, which has previously been associated with AEP [4]. Although tigecycline has high tissue penetration and lipophilicity [1], whether these pharmacologic properties contribute to pulmonary antigen presentation or eosinophilic inflammation remains hypothetical and cannot be determined from the present case.

Although mild bilateral pulmonary opacities were present before tigecycline initiation, serial pre‐treatment chest radiographs showed predominantly low‐volume and bibasilar atelectatic changes. The patient remained asymptomatic without hypoxaemia, fever or other respiratory manifestations despite prolonged hospitalisation following major trauma. After 21 days of tigecycline therapy, he developed acute hypoxaemic respiratory failure accompanied by marked radiographic progression with new diffuse bilateral infiltrates and ground‐glass opacities, as well as profound BAL eosinophilia. Therefore, the temporal, clinical and radiographic evolution was considered more consistent with a new acute pulmonary process arising during tigecycline exposure than with simple progression of the pre‐existing radiographic abnormalities.

Several concomitant medications were carefully evaluated as potential alternative causes (Table 2). Acetaminophen, vancomycin, cephalexin and levetiracetam have been implicated in rare reports of eosinophilic pneumonia or pulmonary infiltrates with eosinophilia, while ceftriaxone has been associated with drug‐induced hypersensitivity pneumonitis with BAL eosinophilia. However, vancomycin, ampicillin‐sulbactam, ceftriaxone and cephalexin had all been discontinued before the onset of respiratory deterioration. Micafungin and levetiracetam were considered less likely because their timing was not compatible with symptom onset. In contrast, tigecycline was administered continuously from 12 December to 2 January, and the approximately 3‐week interval between treatment initiation and respiratory deterioration was compatible with the latency generally reported for drug‐induced AEP [5, 6, 7, 8, 9]. The temporal relationship with tigecycline exposure, marked BAL eosinophilia and evaluation of alternative aetiologies support tigecycline as the probable causative agent. However, the patient's clinical improvement followed several simultaneous interventions, including tigecycline discontinuation, cefiderocol initiation and corticosteroid therapy. Therefore, the clinical response supports, but does not prove, a causal relationship between tigecycline and AEP. Drug rechallenge was not performed because it was considered clinically inappropriate and potentially unsafe. This case highlights the diagnostic importance of BAL and the need for prompt recognition and discontinuation of a suspected agent. Tigecycline‐associated AEP may be a rare but potentially reversible complication. Clinicians should consider this diagnosis in patients who develop respiratory deterioration during tigecycline therapy, particularly when BAL demonstrates marked eosinophilia despite minimal peripheral eosinophilia.

**TABLE 2:** Medication exposures preceding the onset of acute hypoxaemic respiratory failure.

Medication	Indication	Dose and route	Start date	Discontinuation date
**Tigecycline**	**Multidrug‐resistant wound infection**	**100 mg IV loading dose followed by 50 mg IV every 12 h**	**12 Dec**	**2 Jan**
Acetaminophen	Fever or pain	1 g IV every 6 h as needed	10 Nov	30 Jan
Levetiracetam	Seizure prophylaxis	1 g IV every 12 h	31 Oct	30 Jan
Vancomycin	Cellulitis or pneumonia	1 g IV every 8 h	4 Nov	8 Nov
Vancomycin	Cellulitis or pneumonia	1 g IV every 8 h	9 Dec	11 Dec
Vancomycin	Cellulitis or pneumonia	1 g IV every 8 h	30 Dec	31 Dec
Ceftriaxone	Pneumonia	2 g IV every 24 h	8 Oct	8 Oct
Ceftriaxone	Pneumonia	2 g IV every 24 h	9 Dec	10 Dec
Cephalexin	Cellulitis	500 mg PO four times daily	5 Nov	12 Nov
Ampicillin–sulbactam	Pneumonia	3 g IV every 6 h	8 Oct	15 Oct
Ampicillin–sulbactam	Pneumonia	3 g IV every 6 h	11 Dec	12 Dec
Micafungin	Empiric antifungal therapy	100 mg IV every 24 h	12 Dec	22 Jan
Duloxetine	Depression	20 mg PO twice daily	11 Nov	30 Jan
Gabapentin	Neuropathic pain	125 mg PO three times daily	9 Nov	31 Jan
Sertraline	Depression	50 mg PO once daily	31 Oct	23 Nov
Ketorolac	Pain	15 mg IV as needed	9 Nov	9 Nov
Silver sulfadiazine	Burn or wound care	1% cream applied topically twice daily	21 Nov	9 Dec
Aluminium hydroxide–magnesium hydroxide–simethicone	Dyspepsia	5 mL PO every 6 h as needed	2 Dec	31 Dec
Dicyclomine	Abdominal cramping	20 mg PO every 6 h as needed	12 Dec	31 Dec
Diphenhydramine	Pruritus	50 mg PO per dose; two doses administered	31 Oct	23 Nov
Hydromorphone	Pain	0.5 mg IV as needed	23 Oct	5 Dec
Ephedrine sulphate	Hypotension	5 mg IV once	12 Dec	12 Dec
Fentanyl	Analgesia	50 mcg IV once	20 Oct	20 Oct
Fentanyl	Analgesia	50 mcg IV once	12 Dec	12 Dec
Glycopyrrolate	Secretion control	0.2 mg IV once	11 Oct	11 Oct
Glycopyrrolate	Secretion control	0.2 mg IV once	12 Dec	12 Dec
Insulin glargine	Hyperglycaemia	12–25 units SC daily	6 Oct	30 Jan
Iopamidol	Contrast‐enhanced imaging	1.5–2 mL/kg IV once	14 Nov	14 Nov
Iopamidol	Contrast‐enhanced imaging	1.5–2 mL/kg IV once	9 Dec	9 Dec
Lidocaine	Local anaesthesia for procedure	20 mg/mL solution administered subcutaneously	12 Dec	12 Dec
Loperamide	Diarrhoea	4 mg PO once	20 Nov	20 Nov
Mafenide	Burn or wound care	8.5% cream applied topically	9 Oct	9 Dec
Methadone	Pain	17.5 mg PO every 8 h	3 Nov	Ongoing at discharge
Metoclopramide	Promotion of gastrointestinal motility	10 mg IV every 8 h	12 Dec	Ongoing at discharge
Midazolam	Sedation	1 mg IV as needed	20 Oct	26 Jan
Midodrine	Hypotension	2.5 mg PO every 8 h	7 Dec	6 Jan
Ondansetron	Nausea and vomiting	4 mg IV as needed	6 Oct	25 Jan
Oxycodone	Pain	10 mg PO every 4 h as needed	2 Nov	8 Jan
Oxycodone–acetaminophen	Pain	10/325 mg PO every 4 h as needed	6 Oct	12 Nov
Pantoprazole	Stress‐ulcer prophylaxis	40 mg PO every morning	7 Oct	24 Jan
Propofol	Sedation during mechanical ventilation	5–50 mcg/kg/min IV infusion	1 Oct	3 Oct
Rocuronium	Neuromuscular blockade for procedure	100 mg IV once	12 Dec	12 Dec
Scopolamine	Secretion control	One transdermal patch every 72 h	12 Dec	31 Dec
Sodium hypochlorite	Burn or wound care	0.5% solution applied topically to the wound	12 Dec	12 Dec
Sugammadex	Reversal of neuromuscular blockade	100 mg IV once	12 Dec	12 Dec
Temazepam	Insomnia	15 mg PO nightly	30 Oct	27 Dec

*Note:* Medications administered during the relevant review period were evaluated to identify potential alternative causes of drug‐induced eosinophilic pneumonia and to assess the temporal relationship between medication exposure and the development of probable tigecycline‐induced acute eosinophilic pneumonia. The medication review focused on the 4–6 weeks preceding the onset of acute hypoxaemic respiratory failure. Medications initiated earlier were included when considered potentially relevant to the assessment of drug‐induced lung injury. Tigecycline is listed first as the suspected causative agent, followed by medications previously reported to be associated with eosinophilic pneumonia or drug‐induced lung injury; these medications are identified by bold. For intermittently administered medications, the start and discontinuation dates indicate the first and last documented administrations.

Abbreviations: IV, intravenous; PO, oral; SC, subcutaneous.

## Author Contributions

A.E. drafted the manuscript; M.K. revised the manuscript and approved the final version.

## Funding

The authors have nothing to report.

## Consent

The authors declare that written informed consent was obtained from the patient's legally designated power of attorney for the publication of this manuscript and accompanying images and attest that the form used to obtain consent from the patient complies with the journal requirements as outlined in the author guidelines.

## Conflicts of Interest

The authors declare no conflicts of interest.

## Data Availability

The data that support the findings of this study are available on request from the corresponding author. The data are not publicly available due to privacy or ethical restrictions.
